# A non-internalised CD38-binding radiolabelled single-domain antibody fragment to monitor and treat multiple myeloma

**DOI:** 10.1186/s13045-021-01171-6

**Published:** 2021-11-02

**Authors:** Elodie Duray, Margaux Lejeune, Frederic Baron, Yves Beguin, Nick Devoogdt, Ahmet Krasniqi, Yoline Lauwers, Yong Juan Zhao, Matthias D’Huyvetter, Mireille Dumoulin, Jo Caers

**Affiliations:** 1 Laboratory of Haematology, GIGA-I3, University of Liège, Liège, Belgium; 2 Department of Medical Imaging, Laboratory for In Vivo Cellular and Molecular Imaging, Vrije Universiteit Brussel, Brussels, Belgium; 3 School of Chemical Biology and Biotechnology, University Shenzhen Graduate School, Peking, China; 4 NEPTUNS, Nanobodies To Explore Protein Structure and Functions, Centre for Protein Engineering (CIP), University of Liège, Liège, Belgium; 5 Division of Haematology, Department of Medicine, University and CHU of Liège, Liège, Belgium

**Keywords:** Multiple myeloma, CD38, Theranostic, Single-domain antibody, Nanobody, Radio-immunotherapy, Targeted radionuclide therapy

## Abstract

**Background:**

Antibody-based therapies targeting CD38 are currently used as single agents as well as in combination regimens for multiple myeloma, a malignant plasma cell disorder. In this study, we aimed to develop anti-CD38 single-domain antibodies (sdAbs) that can be used to trace CD38^+^ tumour cells and subsequently used for targeted radionuclide therapy. SdAbs are derived from *Camelidae* heavy-chain antibodies and have emerged as promising theranostic agents due to their favourable pharmacological properties.

**Methods:**

Four different anti-CD38 sdAbs were produced, and their binding affinities and potential competition with the monoclonal antibody daratumumab were tested using biolayer interferometry. Their binding kinetics and potential cell internalisation were further studied after radiolabelling with the diagnostic radioisotope Indium-111. The resulting radiotracers were evaluated in vivo for their tumour-targeting potential and biodistribution through single-photon emission computed tomography (SPECT/CT) imaging and serial dissections. Finally, therapeutic efficacy of a lead anti-CD38 sdAb, radiolabelled with the therapeutic radioisotope Lutetium-177, was evaluated in a CD38^+^ MM xenograft model.

**Results:**

We retained anti-CD38 sdAb #2F8 as lead based on its excellent affinity and superior stability, the absence of competition with daratumumab and the lack of receptor-mediated internalisation. When intravenously administered to tumour-xenografted mice, radiolabelled sdAb #2F8 revealed specific and sustained tumour retention with low accumulation in other tissues, except kidneys, resulting in high tumour-to-normal tissue ratios. In a therapeutic setting, myeloma-bearing mice received three consecutive intravenous administrations of a high (18.5 MBq) or a low radioactive dose (9.3 MBq) of ^177^Lu-DTPA-2F8 or an equal volume of vehicle solution. A dose-dependent tumour regression was observed, which translated into a prolonged median survival from 43 days for vehicle-treated mice, to 62 days (*p* = 0.027) in mice receiving the low and 65 days in mice receiving the high (*p* = 0.0007) radioactive dose regimen, respectively.

**Conclusions:**

These results highlight the theranostic potential of radiolabelled anti-CD38 sdAbs for the monitoring and treatment of multiple myeloma.

**Supplementary Information:**

The online version contains supplementary material available at 10.1186/s13045-021-01171-6.

## Introduction

The field of theranostics represents the clinic’s ongoing efforts to develop more specific and individualised therapies for various diseases and to combine diagnostic and therapeutic capabilities into a single pharmaceutical agent [1]. Nuclear medicine is ideally positioned to play a central role in theranostics. Indeed, diagnostic radionuclides linked to an antigen-binding vector allow to visualise molecular targets, to provide non-invasive information on biomarker expression, to select patients for targeted therapies and to monitor therapy responses. Conjugating that same vector to therapeutic radionuclides enables targeted radionuclide therapy (TRNT).

Immunotherapy is revolutionising the treatment of multiple myeloma (MM). This disease, characterised by the clonal proliferation of malignant plasma cells in the bone marrow, has a high unmet therapeutic need [2]. Daratumumab is the first-in-class CD38-binding monoclonal antibody (mAb) that is currently FDA- and EMA-approved for monotherapy or combination therapy for relapsed MM, based on impressive results in large phase 3 trials [3]. Unfortunately, not all pre-treated patients respond to daratumumab, and some patients who initially respond eventually become resistant to the treatment. Although baseline CD38 levels are predictive of response, they are not routinely evaluated before the initiation of daratumumab treatment. Therefore, there is a need for theranostic agents to identify and treat patients who might benefit from anti-CD38-based therapies.

Different CD38-targeting agents have been integrated in theranostic constructs including mAbs, single-chain variable fragments (scFv) and single-domain antibodies (sdAbs) [4–7]. They were either directly or indirectly coupled to diagnostic or therapeutic radionuclides, or fluorochromes. Single-domain antibody fragments (sdAbs) are derived from *Camelidae* heavy-chain antibodies and have emerged as promising vectors [8]. Due to their small size, sdAbs have favourable pharmacological properties compared to conventional Abs or Ab fragments, including an improved tissue penetration, a fast clearance from the circulation and a high conformational stability (Additional file 1: Fig. 1). Moreover, their straightforward production and engineering allow researchers to adapt and include them into a variety of applications [9–11]. Finally, their long flexible antigen-binding loops allow the recognition of buried epitopes, and they appear not to be immunogenic [12].

**Fig. 1:**
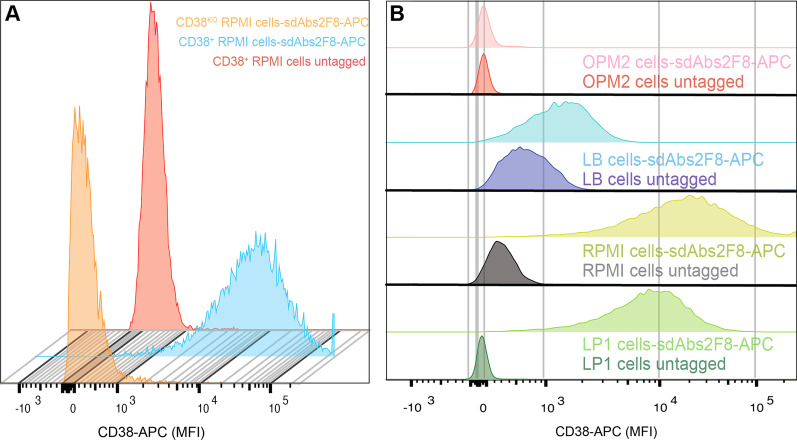
Monoparametric representations of labelled tumour cells with APC from flow cytometry experiments. Image obtained from density plot (FSC/SSC) with single marker APC. **a** Histogram of the mean fluorescence intensity measured on RPMI cells’ surface. The red peak corresponds to RPMI 8226 stained with the secondary APC-labelled anti-H_6_ mAb alone. The blue one shows the staining of RPMI 8226 with sdAb #2F8 followed by the secondary Ab and the orange peak a similar staining on CD38^KO^ cells. **b** Histograms of the 2F8’s association to the surface of different cell lines expressing (LP1, RPMI, LB5871-LYMP (LB) or not (OPM2) the CD38 receptor. A clear binding of the sdAb #2F8 to the surface of LP1 (light green) and RPMI cells (yellow) is observed. SdAb #2F8 also binds to the surface of lymphoma cells, although less significantly (LB, light blue). No interactions were identified on the OPM2’s membrane (red peaks)

In this work, we describe the development of a radiolabelled sdAb as a theranostic agent targeting CD38 that could ultimately predict responsiveness to and at the same time allow for an anti-CD38 treatment strategy.

## Material and methods

### Expression and purification of His-tagged and untagged sdAbs

Non-targeting control sdAbs cAb-BcII10 and R3B23 were generated as described before [13]. To obtain the four hexahistidine (His_6_)-tagged sdAbs (i.e. #551 [5], #375 [5], #1053 [5] and #2F8 [14]), their genes were cloned into the expression vectors pHEN2 or pHEN6 and subsequently transformed into the *E. coli* WK6 cells [15]. These His-tagged sdAbs were purified by metal chelate affinity chromatography [10]. Residual imidazole was removed by gel filtration (Sephadex G25). A stop-codon was introduced by mutation before the His-tag coding region to produce untagged nanobodies #551 and #2F8. Untagged sdAbs were produced and subsequently purified by combining ionic exchange (high TRAP Q HP and Capto S resin) and size exclusion chromatography (Superdex 75) in 50 mM HEPES, 150 mM NaCl. The purity and integrity of sdAbs were evaluated by SDS-PAGE and by mass spectrometry analysis.

### Characterisation of anti-CD38 sdAbs

#### Flow cytometry

The specific binding of sdAbs to cells expressing CD38 was assessed using different cancer cell lines, such as RPMI 8226 cells (CD38^+^ or CD38^KO^), LP1, K562, U266 and LB5871-LYMP. Cells were incubated with 100 nM of purified His_6_-tagged sdAbs for 30 min at 4 °C. After washing with PBS-3%FBS, the cells were incubated with a secondary antibody (APC-conjugated anti-His mAb, BioLegend). Stained cells were analysed on a BD FACSArray™ Bioanalyzer System (BD Biosciences). An irrelevant non-targeting sdAb (cAb-BcII10) was used as a reference for non-specific binding. To study the internalisation of sdAb #2F8, the same cells were incubated with 10 nM sdAb 2F8 at 37 °C over time. At different time points during incubation, cells were labelled with the secondary anti-His mAb to measure the dynamics of sdAbs bound to the cell membrane.

#### Biolayer interferometry

To assess the binding affinity by interferometry using the OctetHTX platform, streptavidin (SA)-coated biosensor tips (Pall ForteBio) were used to capture the biotinylated extracellular domain of the CD38. Kinetic measurements for sdAb binding were performed by dipping the CD38-coated biosensors into wells containing seven different concentrations of the corresponding sdAbs (from 2.5 to 50 nM), followed by a dissociation time by transferring the biosensors into buffer-containing wells. All sensorgrams were referenced for buffer effects and then fitted using the OctetHTX user software. Kinetic responses were fitted to a 1:1 binding model to obtain values for association (k_on_) and dissociation (k_off_) rate constants, and the equilibrium dissociation constant (K_D_). This experiment was also performed for DTPA-conjugated #2F8 but with four different concentrations (from 20 to 100 nM). Competition between antibodies for CD38 binding was assessed by dipping the CD38-coated biosensors into wells containing a first antibody of interest (100 nM) until saturation (first association). Then, the sensors were immersed into a well containing the first and second antibodies (100 nM) (second association) allowing to monitor the binding capacities of the second antibody in the presence of the first one.

#### Protein stability

Circular dichroism (CD) measurements were taken with a 810 Jasco Spectrophotometer, either in the far UV (190–250 nm) or in the near UV (250–350 nm) regions, using a sdAb concentration of 0.2 mg/mL in 50 mM sodium buffer, and 0.1-cm or 1-cm pathlength quartz cell, respectively. Spectra were acquired at a scan speed of 50 nm/min, with a 1-nm bandwidth, a 2-s integration time and at 25 °C. Five spectrums were acquired, averaged and corrected by subtracting the buffer spectrum recorded under identical conditions. Thermal unfolding experiments were monitored at 205 nm by raising the temperature from 25 to 97 °C at a rate of 0.5 °C/min. The reversibility of the transition was assayed by cooling the sample down to 25 °C. Data were acquired with a 4-s integration time and a 1-nm bandwidth. The heat-induced unfolding transitions monitored were used to determine the mid-transition temperature (T_m,_ temperature at which 50% of the protein molecules are unfolded, while the remaining are native) by fitting the experimental data with the equation of a two-state model [16]. Since the transitions were not reversible, only apparent Tm could be determined (Tm*).

### Cell line and mouse model

The human MM cancer cell line RPMI 8226 (GFP^+^/Luciferase^+^) was obtained from A. Martens (Haematology department, VU University Medical Center, The Netherlands). RPMI 8226 cells were cultured using Roswell Park Memorial Institute medium (RPMI 1640, Lonza, Belgium) enriched with 10% foetal bovine serum, 100 U/mL penicillin and 0.1 mg/mL streptomycin. K562 cells were purchased from ATCC (Manassas, Virginia, US), double-hit lymphoma cell line LB5871-LYMP was obtained from N. Van Baren (UCL, Brussels) [17], and finally, U266, OPM2 and LP-1 cell lines were obtained from H. Jernberg-Wiklund (Uppsala University, Sweden), respectively.

The CD38 knockout (KO) cells used as specific controls were obtained by genetic engineering using the CRISPR/Cas9 technique as previously described [18].

Tumour xenografts were obtained by subcutaneously (s.c.) inoculating 6- to 12-week-old NOD.Cg-Prkdc^sci^ Il2rg^tm1Wjl^^1^wʲˡ / SzJ (NSG) mice with RPMI 8226 cells (0.5 × 10^6^) in 150 μL PBS-Matrigel (ratio 1:1) into the left hind. Mice were irradiated with 2 Gy 24 h before cell injection to favour a homogenous tumour uptake. Tumour development was followed from day 14 (palpable from day 19) using an IVIS Spectrum In Vivo Imaging System (PerkinElmer). Briefly, after s.c. injection of D-luciferin (150 mg/kg), anaesthetised mice were imaged (scan and reading of luminescence emitted during substrate degradation by cancer cells expressing the luciferase). All animal experiments were approved by the Ethical Committee for Animal Experiments of the University of Liège and of Vrije Universiteit Brussel.

### Nuclear imaging using ^99m^Tc-sdAbs

Anti-CD38 sdAbs were radiolabelled with ^99m^Tc at their C-terminal His_6_-tag using the Isolink labelling kit (Mallinckrodt Medical BV), as previously described [19]. ^99m^Tc-sdAbs were separated from unreacted [^99m^Tc(H_2_O)_3_(CO)_3_]^+^ via NAP-5 size exclusion chromatography (Sephadex, GE Healthcare). The eluate was passed through a 0.22 μm filter (Millipore), and radiochemical purity (RCP) was determined by instant thin-layer chromatography (iTLC) using acetone as running buffer (iTLC-SG, Pall Corporation) and was > 95%. Mice bearing s.c. CD38^+^ RPMI 8226 tumours (n = 3) were intravenously (i.v.) injected with ^99m^Tc-labelled sdAbs (about 4 μg and 51.1 ± 3.7 MBq). At 1 h post-injection (p.i.), mice were imaged using pinhole single-photon emission computed tomography (SPECT)/ computed tomography (CT) with a Vector + /CT MILabs system [20]. Images were obtained using a rat SPECT-collimator (1.5-mm pinholes) in spiral mode, 7 positions with 128 s per position for whole-body imaging. Images were reconstructed with 0.4 mm^3^ voxels with 2 subsets and 2 iterations, without post-reconstruction filter. For CT, a normal scan mode of two positions was used. Images were fused and corrected for attenuation based on the CT scan. Image analysis was performed using a Medical Image Data Examiner (AMIDE) software and data expressed as % injected activity per cm^3^ (% IA/cm^3^) [21]. After imaging, mice were killed, and organs and tissues were isolated and weighed. The radioactivity in each sample was measured using a Wizard2 γ-counter (PerkinElmer). Tracer uptake was expressed as % injected activity per gram organ (% IA/g).

### Preparation of ^111^In- and ^177^Lu-DTPA-sdAbs

Untagged #2F8 and R3B23 (3 mg/mL) reconstituted in 50 mM sodium carbonate buffer pH 8.5 were reacted with a tenfold molar excess of bifunctional chelator CHX-A’’-DTPA (Macrocyclics) for 3 h at RT, as described previously [22]. The conjugation reaction was quenched by reducing the pH of the mixture to pH 7.0. DTPA-2F8 was purified on Superdex 75 10/300 GL (GE Healthcare) in 0.1 M ammonium acetate buffer pH 7.0. The necessary amount of ^111^In (54 to 270 MBq) or ^177^Lu (74 MBq to 1 GBq) was incubated for 30 min at RT or 37 °C, respectively, with the DTPA-2F8 in 200 mM ammonium acetate pH 5. Resulting ^111^In- and ^177^Lu-DTPA-2F8 were purified on NAP-5 column (GE Healthcare) using 0.9% NaCl as eluent. In case of high radioactive labelling for preclinical therapy, 0.9% NaCl with 5 mg/mL ascorbic acid was used. The resulting radio-conjugates were filtered on 0.22 μm after which RCP was determined by iTLC with citric acid as running buffer (iTLC-SG, Pall Corporation) and measured > 96%.

### In vitro evaluation of ^111^In-DTPA-2F8

Binding affinity and degree of internalisation of ^111^In-DTPA-2F8 were evaluated as previously described [23]. In case of assessing binding affinity, CD38^+^ RPMI 8226 cells were incubated for 1 h at 4 °C with a serial dilution of ^111^In-DTPA-2F8 (0.1–300 nM) alone or in combination with a 100-fold molar excess of unlabelled 2F8 to assess non-specific binding. Next, unbound sdAb was washed away, after which retained radioactivity was measured in a γ-counter. Data were plotted using GraphPad Prism software. To study the level of receptor-mediated internalisation, RPMI 8226 cells were incubated for 1 h at 4 °C with 10 nM of ^111^In-DTPA-2F8, alone or in combination with a 100-fold molar excess of unlabelled #2F8 to assess unspecific binding. After washing, the cells were incubated at 37 °C up to 24 h. At different time points during incubation, cells were processed to obtain the corresponding dissociated, membrane-bound and internalised fraction, as described before [23]. All fractions were measured for radioactivity in a γ-counter.

### Nuclear imaging using ^111^In-DTPA-sdAbs

Mice bearing s.c. CD38^+^ RPMI 8226 tumours (n = 3) were i.v. injected with ^111^In-DTPA-2F8 (about 18.8 ± 0.1 MBq) or R3B23 and 150 mg/kg gelofusin. SPECT images were taken at different time points up to 48 h p.i. Images were obtained using the same scan parameters used as described for ^99m^Tc acquisitions, except for the number of positions [6] and time per position for the full body imaging (150 s) and number of iterations (6 instead of 7). After the last image acquisition, mice were killed and processed as described above. In parallel, a few animals (n = 3) were i.v. injected (4.5 ± 0.2 MBq) with ^111^In-DTPA-2F8 or ^111^In-DTPA-R3B23, alone or as a co-injection with 150 mg/kg gelofusin. They were imaged 1 h post-injection in order to evaluate the effect of gelofusin on the renal retention of the tracer.

### Biodistribution and dosimetry ^177^Lu-DTPA-2F8

Mice bearing CD38^+^ RPMI 8226 tumours were i.v. injected with ^177^Lu-DTPA-2F8 (about 4 μg and 4.5 ± 0.2 MBq) co-infused with 150 mg/kg gelofusin (n = 3). Next, mice were killed at different time points up to 48 h p.i., followed by the isolation of different organs and tissues. All samples were weighed and counted for radioactivity content against a standard of known radioactivity using a γ-counter and expressed as percentage of the injected activity per gram of tissue mass (% IA/g), corrected for decay. For dosimetry purposes, the biodistribution data were time-integrated to obtain the residence time per gram tissue [22]. Briefly, the area under the curve between 1 and 48 h was made using the trapezoid integration method. Next, the absorbed doses were calculated using S values for ^177^Lu obtained from RADAR phantoms (Unit Density Spheres).

### Targeted radionuclide therapy using ^177^Lu-DTPA-2F8

Day 23 post-cell inoculation, mice with palpable CD38^+^ RPMI 8226 tumours were randomly categorised into three treatment groups (n = 10). Mice received three consecutive i.v. administrations, once every two days, of either: (i) a high radioactive dose (about 18.5 ± 0.5 MBq), (ii) a low radioactive dose (9.3 ± 0.3 MBq) ^177^Lu-DTPA-2F8 + 150 mg/kg gelofusin, or (iii) an equal volume of vehicle solution (PBS). Animals were followed up over time through body condition scoring (weight, physical appearance, mobility and behaviour), and tumour volume was assessed via calliper measurements and bioluminescence imaging as described above.

## Results

### In vitro characterisation of sdAbs

**Fig. 2:**
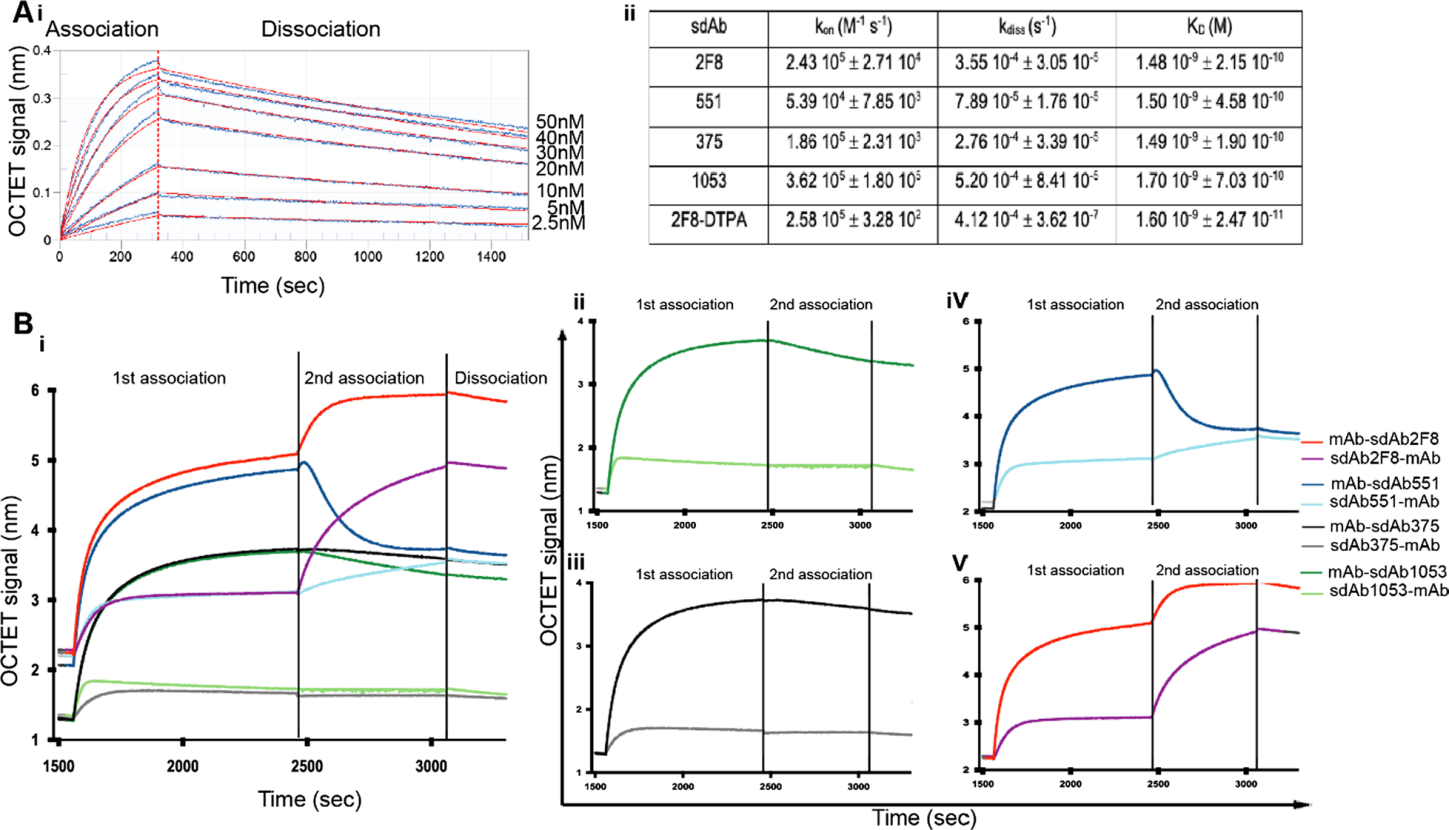
**a** (i) Biolayer interferometry sensorgrams from a dilution series of sdAb #2F8 from 50 to 2.5 nM to assess its binding to the CD38. The blue curves represent the experimental kinetics, and the red ones represent fit curves. (ii) Summary of the different binding parameters for the sdAbs studied and the sdAb 2F8 conjugated to the chelator DTPA used in radiolabelling with ^111^In and ^177^Lu. The data are expressed as mean ± SD (n = 4). **b** (i) Competitive binding between daratumumab and sdAbs (#2F8, #551, #375 or #1053) for binding to the CD38, (ii) data obtained for competitor sdAb #1053 and daratumumab, where no additional signal is observed upon addition of sdAb on mAb-CD38 complex (dark green) and vice versa (light green), (iii) similar results obtained for the sdAb #375 (addition of sdAb on mAb-CD38 complex in black and the reverse in grey), (iv) partial-competitor sdAb #551 and daratumumab where an additional signal is observed upon the addition of mAb on sdAb-CD38 complex but with a lower amplitude than when it was immobilised first (light blue). A decrease in signal after addition of sdAb on mAb-CD38 complex is observed (dark blue), which may indicate loss of binding for daratumumab. Finally, (v) non-competitor sdAb #2F8 and daratumumab, where no competition is observed. The two proteins are able to bind the receptor without impacting the signal amplitude, regardless of the order of addition

Flow cytometry experiments revealed the specific binding of the different anti-CD38 sdAbs. Indeed, all of them are capable of binding the CD38 antigen on MM cell lines (RPMI 8226 and LP1) and non-Hodgkin’s lymphoma cells (LB5871-LYMP), while no specific binding was observed on the CD38^−^ cells (K562 cell line) and CD38^KO^ cell lines (Fig. 1). Non-targeting cAb-BcII10 did not show specific binding to CD38. 2F8 binds CD38 with an affinity in the low (K_D_ 1.5 nM) nanomolar range as illustrated in Fig. 2. Apparent midpoint of the heat-induced unfolding (Tm*), ranged from 69 to 89 °C, were obtained for the studied sdAbs with the #2F8 protein showing the highest one (88.7 ± 0.3 °C). To determine potential competition for CD38 targeting between daratumumab and the different sdAbs, competitive biolayer interferometry (BLI) experiments were performed. Different levels of competition with daratumumab were defined: total, partial or null. No binding of sdAbs #1053 or #375 was detected on CD38 that was pre-incubated with daratumumab, and vice versa. In contrast, sdAb #2F8 still bound in full capacity, and sdAb #551 in part, to the extracellular domain of CD38 in the presence of daratumumab (Fig. 2). The set of data acquired for the other three sdAbs are summarised in Table 1.

**Table 1:** Summary of in vitro and in vivo characterisation of the different anti-CD38 sdAbs. The affinities and competition behaviour towards the human monoclonal antibody daratumumab were obtained by the biolayer interferometry method

Parameters sdAbs	#375	#1053	#551	#2F8
Affinity (K_D_)	1.49 nm	1.7 nM	1.50 nM	1.48 nM
Competition Vs daratumumab	Competitor	Competitor	Partial competitor	Non-competitor
Biodistribution				
Location	ND	1.78 ± 0.37	3.37 ± 0.38	2.22 ± 0.47
Tumour	ND	258.00 ± 19.08	402.20 ± 22.46	169.32 ± 4.56
Blood	ND	0.61 ± 0.26	1.33 ± 0.08	0.53 ± 0.12
Liver	ND	1.28 ± 0.28	1.33 ± 0.08	0.53 ± 0.12
Thermal stability (Tm*)	69.1 ± 0.1 °C	73.1 ± 0.1 °C	72.2 ± 0.2 °C	88.7 ± 0.3 °C

**Fig. 3:**
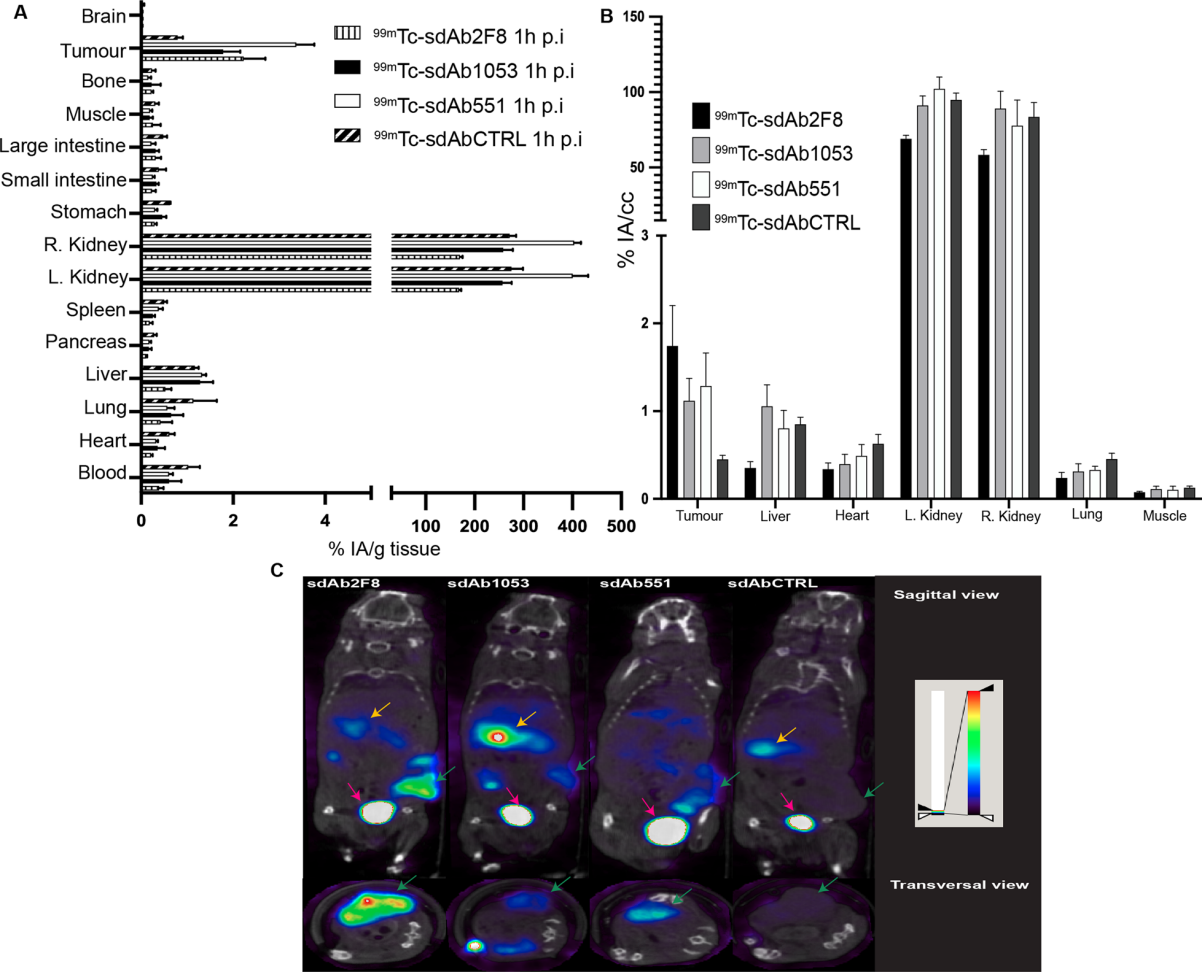
**a** Ex vivo biodistribution of ^99m^Tc-labelled sdAbs in RPMI 8226 CD38^+^ tumour-bearing mice based on dissection data at 1 h p.i. and expressed as % IA/g (data from these results are included in Additional file 1: Table 1). **b** In vivo biodistribution of the radiolabelled sdAbs injected in mice expressed as % IA/cc. Data shown in Additional file 1: Table 3. Data are mean ± standard deviation of three mice/sdAb. A radiolabelled irrelevant non-targeting sdAb was used as negative control. **c** Sagittal and transversal view on fused whole-body and tumour-focused μSPECT/CT acquisitions 1 h p.i. of intravenously injected ^99m^Tc-H_6_-2F8, ^99m^Tc-H_6_-1053, ^99m^Tc-H_6_-551 and non-targeting ^99m^Tc-H_6_-CTRL in MM mice. One representative of each group is shown. Green arrows indicate tumour uptake, while the yellow and pink arrows indicate renal and urinary bladder uptake, respectively. NIH + white colour scale is used, and images are normalised to the activity at the time of acquisition and equally scaled down to 4% relative to maximum activity in image. For all CD38 sdAbs, the following observation can be made: a non-specific uptake by the kidneys, a tumour site-specific accumulation and a background radioactivity level in most vital organs, albeit at different levels depending on the sdAb. Tumour-to-organ ratios were calculated and are described in Additional file 1: Table 2

All the sdAbs have an affinity in the nanomolar range. #375 and #1053 are in competition with daratumumab, while a partial and no competition are observed for #551 and #2F8, respectively. The data included in the biodistribution section correspond to the results obtained after radiolabelling of sdAbs with the ^99m^Tc radioisotope (Fig. 3); #551 and #2F8 show the best tumour uptake. The thermal stability (Tm*) of the sdAbs was evaluated by far UV circular dichroism. The most stable is #2F8. Based on these characteristics, sdAb 2F8 was chosen for further work. (ND: not-determined, protein not studied in vivo because its in vitro behaviour is similar to that of sdAb 1053)

### Biodistribution of ^99m^Tc -labelled sdAbs

The biodistribution and tumour-targeting potential of different ^99m^Tc-labelled sdAbs were assessed via pinhole μSPECT/CT imaging and dissection. The resulting uptake values in tumours and non-target organs are summarised in Table 1. Administration of ^99m^Tc-H_6_-2F8 revealed a tumour uptake of 2.22 ± 0.47% IA/g compared to only 0.79 ± 0.11% IA/g for ^99m^Tc-H_6_-cAb-BCII10, with good contrasts observed early after tracer administration (Fig. 3). In addition, ^99m^Tc-H_6_-2F8 was found concentrated in kidneys and bladder, in line with the fast blood clearance of sdAbs [24]. Only low amounts of radioactivity were measured in blood (0.39 ± 0.09% IA/g) and non-targeted organs and tissues (from 0.02 ± 0.01 to 0.53 ± 0.12% IA/g) (Additional file 1: Table 1).

### Saturation binding and degree of internalisation of ^111^In-labelled sdAb 2F8

**Fig. 4:**
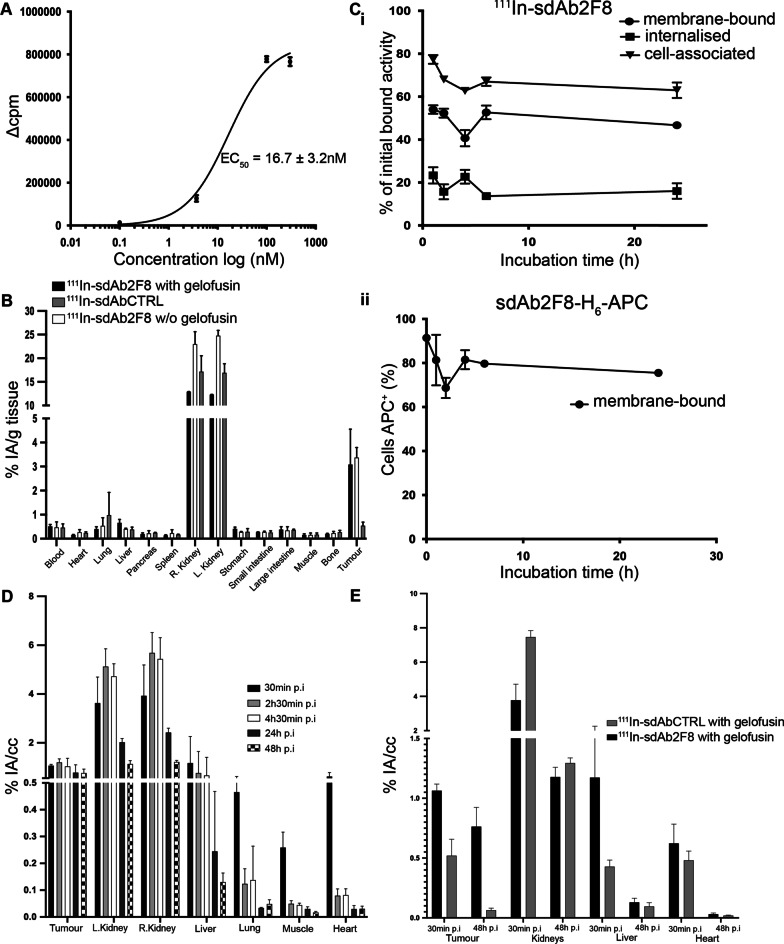
**a** Saturation binding curve of ^111^In-DTPA-2F8 on RPMI 8226 cells. The maximal effective concentration (EC_50_) was calculated by subtracting non-specific-bound activity (assessed by adding an excess of unlabelled sdAb) from total bound activity and plotted in function of ^111^In-sdAb concentrations. Data are expressed as mean ± SD. **b** Ex vivo biodistribution of ^111^In-DTPA- 2F8 or R3B23 sdAbs. NOD scid gamma mice (n = 3) bearing RPMI 8226 tumour (10^5^ cells injected subcutaneously in the left side) were injected at -1 h with 4.5 ± 0.2 MBq of radiolabelled sdAb and euthanised for organ harvesting. Data displayed on the bar graph are expressed as % IA/g and detailed in Additional file 1: Table 4. **c** Cell-internalisation assays using sdAb #2F8. (i) Plot representing the internalised fraction of ^111^In-radiolabelled sdAb #2F8 over time, (ii) membrane-bound fraction of APC-anti-Histag-2F8 over time, assessed via flow cytometry. **d** In vivo biodistribution of ^111^In-labelled sdAb #2F8 (18.8 ± 0.1 MBq) + 150 mg/kg gelofusin for a selection of organs and tissues up to 48 h p.i. **e** Biodistribution of ^111^In-labelled sdAb #2F8 and the non-targeting sdAb after 1 and 48 h post-tracer administration (18.8 ± 0.1 MBq). Both are co-injected with 150 mg/kg gelofusin. Data are presented as mean ± SD (n = 3) and further detailed in Additional file 1: Tables 5 and 6. Images illustrating these biodistributions are included in Additional file 1: Fig. 2

Based on the superior in vitro and in vivo characteristics, sdAb #2F8 was selected as lead CD38-targeting compound (Table 1). Untagged #2F8 was conjugated to p-SCN-Bn-CHX-A”-DTPA and was shown to maintain its binding characteristics as determined with radioactivity and BLI experiments (Figs. 2 and 4). After radiolabelling with ^111^In, both the maximal effective concentration (EC_50_) and the level of internalisation were assessed on RPMI 8226 cells. EC_50_ for ^111^In-DTPA-2F8 measured 16.7 ± 3.2 nM. ^111^In-DPTA-2F8 bound well to CD38 + RPMI 8226 cells but did not induce receptor-mediated internalisation, as this fraction remained low at all time points until 24 h (Fig. 4, panel Ci). Flow cytometry confirmed the minor internalisation of sdAb #2F8 (Fig. 4, panel Cii).

### Biodistribution of ^111^In- and ^177^Lu -labelled sdAb 2F8

**Fig. 5:**
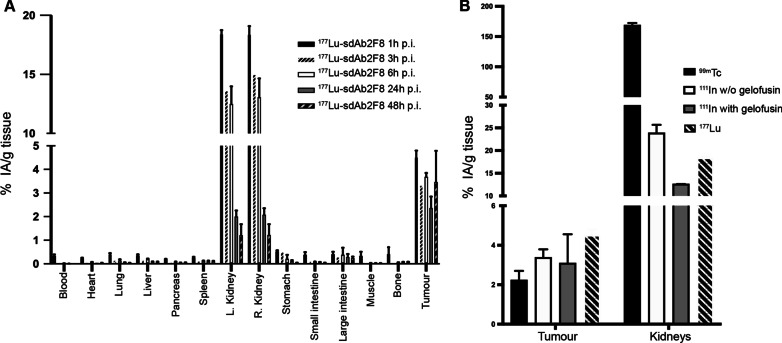
**a** Time-dependent biodistribution of ^177^Lu-DTPA-2F8 i.v. co-injected with gelofusin 150 mg/kg into RPMI 8226 CD38^+^ tumour-bearing mice (10^5^ cells injected subcutaneously in the left back side). Values are represented as % IA/g, obtained after dissection and further detailed in Additional file 1: Table 7. Data are presented as mean ± SD (n = 3). **b** Comparison of renal and tumour retention observed for #2F8 labelled with different radioisotopes (n = 3), alone or with 150 mg/kg gelofusin. Data are expressed as mean ± SD

Mice bearing CD38^+^ RPMI 8226 tumours were i.v. injected with either ^111^In-DPTA-2F8 alone, ^111^In-DPTA-2F8 in combination with 150 mg/kg gelofusin or ^111^In-DTPA-R3B23. ^111^In-DPTA-2F8 showed high and specific uptake in tumour 1 h after tracer injection. High levels of radioactivity were observed in kidneys (23.00 ± 2.62% IA/g) after 1 h, which was reduced by co-injection with 150 mg/kg gelofusin to only 13.00 ± 0.02% IA/g Fig. 4B and Additional file 1: Table 4. ^111^In-DTPA-R3B23 revealed no relevant uptake in tumour (0.54 ± 0.14% IA/g), while the amount in kidneys was similar. Uptake of CD38-specific sdAbs in all other organs and tissues was low and considered as background (Fig. 4). A head-to-head comparison of uptake in tumour and kidneys between ^99m^Tc-H_6_-tagged-2F8, ^111^In- and ^177^Lu-labelled untagged-2F8 is depicted in Fig. 5B. Removal of the hexahistidine tag and co-infusion with 150 mg/kg gelofusin reduced kidney retention by 86 and 57%, respectively. Uptake in tumour of ^177^Lu-DTPA-2F8 co-injected with 150 mg/kg gelofusin measured about 4.5% IA/g and remained constant over time up to 48 h p.i. (Fig. 5A). The renal uptake peaked at 18% IA/g 1 h after administration and decreased to 2% IA/g at 24 h p.i. and about 1.2% IA/g at 48 h p.i. The uptake values over time for different organs and tissues were used for dosimetry calculations. Both tumour and kidneys received the highest absorbed doses per unit of activity, with values of 0.1 and 0.22 Gy per MBq of ^177^Lu-DTPA-2F8 (Additional file 1: **Table 7**). Absorbed doses to additional organs and tissues measured below 0.006 Gy per MBq.

### Therapeutic efficacy of ^177^Lu-DTPA-2F8

**Fig. 6:**
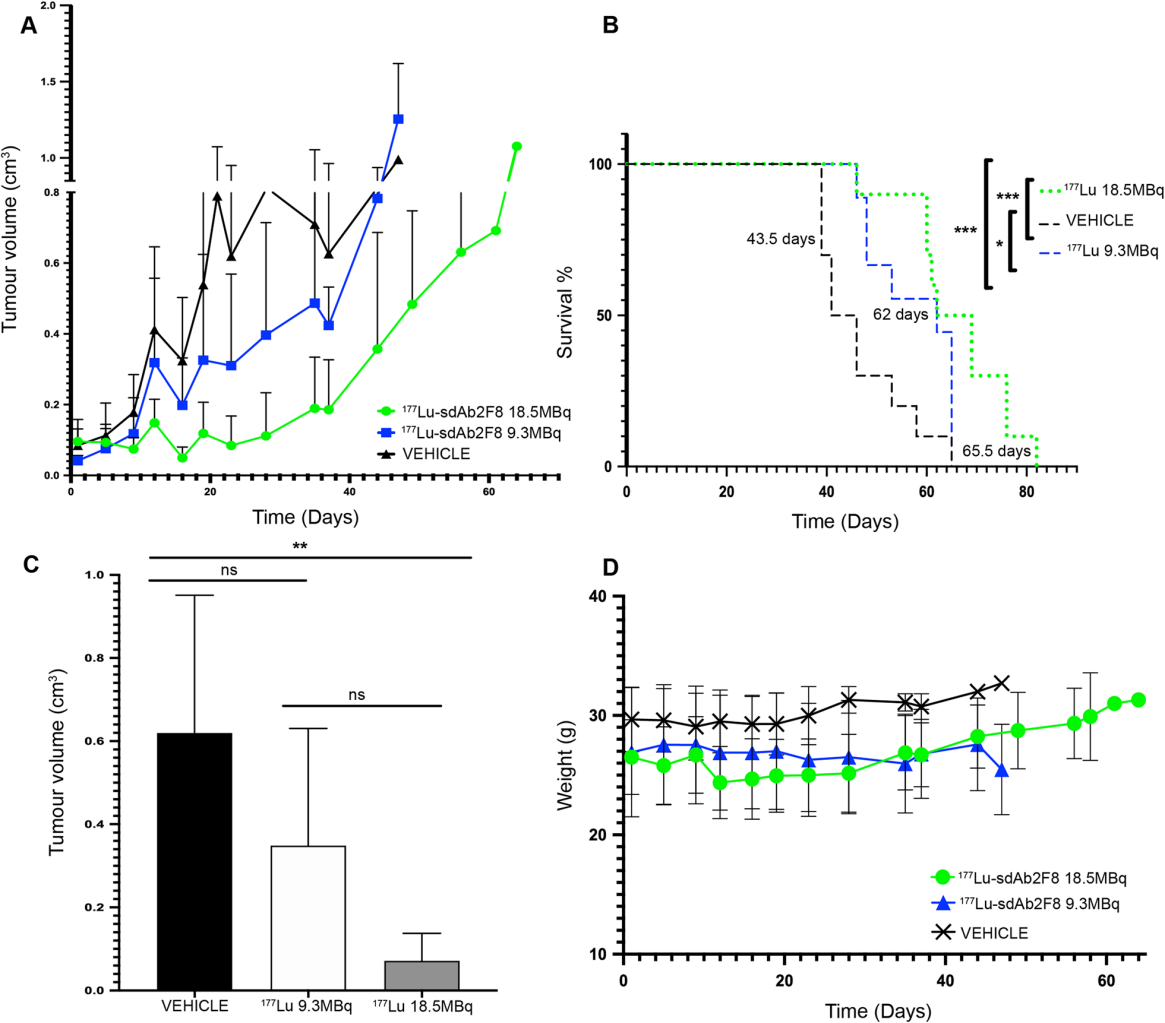
TRNT using ^177^Lu-labelled sdAb 2F8. **a** Tumour volumes (cm^3^) for mice as a function of time (days post-injection), data are expressed as mean ± SD (graph showing the mice individually is presented in Additional file 1: Fig. 3). **b** Resulting survival curves of the different treatment groups (n = 10, tumour inoculation on day 20). Mice injected with vehicle reached tumours > 1 cm^3^ between 21 and 47 days (based on individual analyses). Animals treated with three radioactive doses of 9.3 MBq ^177^Lu-DTPA-sdAb 2F8 had tumours exceeding 1 cm^3^ from day 28 onwards, resulting in a median survival of 62 days post-MM cancer cell inoculation. Animals receiving 18.5 MBq ^177^Lu-DTPA-2F8 three times surpassed a tumour size > 1 cm^3^ from 50 days onwards, and survival was significantly longer (p < 0.0002, log-rank Mantel–Cox test) with a median survival of 65.5 days. Median survival in both groups receiving ^177^Lu-DTPA-2F8 was significantly longer than that for animals receiving vehicle solution (* p < 0.0332, *** p < 0.0002, log-rank Mantel–Cox test). **c** Tumour size comparison at day 23 post-first treatment (or day 43 post-inoculation of MM cancer cells). Data shown are mean ± SD. (ns: not significant, ** p < 0.005, using one-way ANOVA, Tukey’s multiple comparison test). **d** Weight progression of the different treated groups. Data are expressed as mean ± SD

Therapeutic efficacy of ^177^Lu-DTPA-2F8 was assessed in mice bearing CD38^+^ RPMI 8226 tumours. Mice received three i.v. injections of either high radioactive dose (18.5 ± 0.5 MBq) or low radioactive dose (9.3 ± 0.3 MBq) regimen of ^177^Lu-DTPA-2F8, or an equal volume of vehicle solution, which led to cumulative absorbed doses to kidneys and tumour of 6.21 and 2.77 Gy, and 12.31 and 5.50 Gy for the low- and high-dose groups, respectively. Figure 6A illustrates tumour volume evolution for all three treatment groups. The direct comparison of tumour volumes at day 43 after tumour inoculation revealed a dose-dependent tumour reduction for both dose regimens compared to vehicle solution (Fig. 6C). These changes were confirmed by similar reduction in tumour burden, quantified by bioluminescence (not shown). The median survival (Fig. 6B) of vehicle-treated mice was 43 days, compared to 62 days for the low radioactive dose regimen (*p* = 0.027) and 65 days for the high radioactive dose regimen (*p* = 0.0007).

## Discussion

The results presented herein highlight the theranostic potential of radiolabelled CD38-targeting sdAb #2F8 for MM. The radiolabelling of antibodies or Ab-fragments and their subsequent use for nuclear imaging is an attractive tool that helps to assess tumour target expression and distribution in a non-invasive manner. This field of nuclear medicine is dramatically expanding by incorporating new conjugation strategies, radiochemistry procedures and Ab fragments or mimetics. SdAbs, which are the variable binding domains of heavy-chain antibodies, have favourable properties as observed for sdAb #2F8: they have a low molecular weight of about 12–15 kDa and a straightforward recombinant production in different hosts; moreover, they can be easily conjugated with diagnostic or therapeutic isotopes using standardised radiolabelling procedures without affecting protein affinity for its antigen and thus its targeting potential.*Camelidae*

SdAb #2F8 was chosen based on its favourable biodistribution with a good tumour uptake, the absence of competition with daratumumab and the lack of receptor-mediated internalisation. Another CD38-binding sdAb, #1053, was recently conjugated to ^68^ Ga and developed as an imaging probe [25]. Our results indicate that #1053 competes with daratumumab for binding to CD38 and the seminal work of the group of Koch-Nolte showed that both bind to the same epitope [26].

In contrast to daratumumab, which induces an internalisation of the antigen–antibody complex and a further downregulation of CD38 expression on the MM cell [27, 28], sdAb #2F8 is minimally internalised after CD38 binding. The absence of internalisation is an important feature allowing the realisation of a diagnostic immune-PET (positron emission tomography) before a CD38-targeting therapeutic counterpart is given without fearing a reduced antigen expression due to the diagnostic intervention. In addition, as a therapeutic compound, #2F8 can be administrated repeatedly without affecting CD38 expression. The prolonged localisation of #2F8 on the cell membrane favours other therapeutic applications such as pretargeting systems that are based on the separation between the administration of the targeting molecule and the radiolabelled agent [29]. The strong binding, long tumour retention and persistence of its extracellular localisation make this sdAb interesting for further integration in such pretargeting system.

Other vectors targeting CD38 have been reported, with a few strategies already evaluated in the clinic [30, 31]. Except for one bispecific construct, all use full-size mAbs as vector. mAbs are known to have a long blood circulation time and slow accumulation in target tissues which requires to wait days after tracer administration before appropriate image contrast is achieved (for daratumumab, the ideal scanning time is between days 5 and 8) [30]. The fast and specific targeting potential of sdAbs in combination with the fast clearance of the unbound fraction in the kidneys (half-time about 20 min) allows for a same-day imaging approach, much like the current practice for ^18^F-FDG-PET [32–34]. While their reduced size favours fast clearance, the biodistribution studies presented herein indicate, however, a high kidney retention as observed with other sdAbs, peptides or scaffold proteins [35]. This high retention can be explained by their glomerular filtration followed by tubular reabsorption and subsequent lysosomal degradation [35]. Strategies for kidney protection that have been proposed include the infusion of positively charged basic amino acids or gelofusin []. In the presented study, the co-injection of radiolabelled 2F8 with gelofusin decreased renal uptake by 57%.^24^, ^36^

In addition to the diagnostic capacities, #2F8 was also successfully evaluated in the framework of TRNT. Indeed, repeated administration of #2F8, coupled to the β^−^-particle emitting radionuclide ^177^Lu, resulted in a significant decrease in tumour burden and in a prolonged survival of MM-diseased mice. These responses were dose-dependent with a strong reduction with the high radioactive dose regimen. Repeating low radioactive doses resulted in a similar improvement in survival. This treatment strategy was chosen to mimic a clinical approach of fractionated dosing to lower the toxicity on the surrounding healthy tissues, as used for peptide receptor radionuclide therapy with ^177^Lu-DOTATATE [37].

The group of D. Green and O. Press was the first to develop a CD38-based TRNT approach [38]. Both a classical approach using a directly radiolabelled murine anti-CD38 mAb and a more innovative pretargeting approach were assessed. In the first pretargeting study, an antibody-SA construct was combined with a radiolabelled biotin [38]. This pretargeting system improved the radioactivity deposition in organs and tissues and delayed tumour development in a therapeutic setting [38]. In a second pretargeting study, the scFv of an anti-CD38 mAb and a scFV conjugated to Yttrium-DOTA ligand were merged in a bispecific Ab fusion protein [7]. This bispecific Ab showed better anti-tumour effects compared to the SA-biotin system when administered to CD38^+^ tumour-bearing mice. Lastly, an anti-CD38 mAb was radiolabelled with the alpha-particle emitting radionuclide Astatine-211 (^211^At), which also showed to delay tumour growth in MM models with subcutaneous tumours or with minimal residual disease. Other alpha-particle emitters (^212^Pb, ^225^Ac and ^213^Bi) have been evaluated in the framework of CD38-based TRNT and most indicated strong anti-tumour effects [39–41]. One study directly compared the efficacy of mAbs labelled with the α-particle emitting ^225^Ac or with the β^−^-particle emitting ^177^Lu and found a higher efficacy and less toxicity for the ^225^Ac-labelled variant (42).

Radiolabelled sdAbs are promising theranostic vehicles to follow up disease relapse and to treat disseminated disease. The results presented herein highlight the potential of radiolabelled #2F8 to target CD38 expressing MM lesions. Radiolabelled with diagnostic radioisotopes such as ^99m^Tc and ^111^In, #2F8 targets CD38 receptor in a fast and specific manner, allowing high-contrast SPECT images early after tracer administration. Repeated administration of ^177^Lu-labelled #2F8 extends survival of mice significantly compared to controls and in a dose-dependent manner.

## Conclusion

In this study, we identified a new anti-CD38 sdAb fragment that recognises another epitope as the mAb daratumumab and that is not internalised after binding to CD38. This lead sdAb 2F8 had a favourable biodistribution and was successfully integrated in a therapeutic radio-immunoconjugate that delayed tumour progression and prolonged survival of MM xenografts. Taken together, these results highlight the theranostic potential of our lead sdAb and its relevance towards clinical translation.

## Supplementary Information

**Additional file 1.** Supplementary information.

## Data Availability

No datasets were generated in this study. Sequences, plasmids or the sdAb 2F8 can be obtained upon request.

## Abbreviations

sdAb: Single-domain antibody
TRNT: Targeted radionuclide therapy
MM: Multiple myeloma
mAb: Monoclonal antibody
scFv: Single-chain variable fragments
His and H6: Hexahistidine
SA: Streptavidin
CD: Circular dichroism
s.c.: Subcutaneously
i.v.: Intravenously
RCP: Radiochemical purity
iTLC: Instant thin-layer chromatography
BLI: Biolayer interferometry
SPECT: Single-photon emission computed tomography
CT: Computed tomography
PET: Positron emission tomography
99mTc: Technetium-99 m
111In: Indium-111
177Lu: Lutetium-177
211At: Astatine-211
p.i.: Post-injection
% IA/g: Percentage of the injected activity per gram of tissue mass
