# Supplementary material for: A non-internalised CD38-binding radiolabelled single-domain antibody fragment to monitor and treat multiple myeloma
Source: J Hematol Oncol. 2021 Nov 2;14:183. doi: 10.1186/s13045-021-01171-6 (PMC8561907; doi:10.1186/s13045-021-01171-6)
Supplement: Supplementary file 1 — Additional file 1. Supplementary information. [file 13045_2021_1171_MOESM1_ESM.pdf]

Supplementary Figure 1

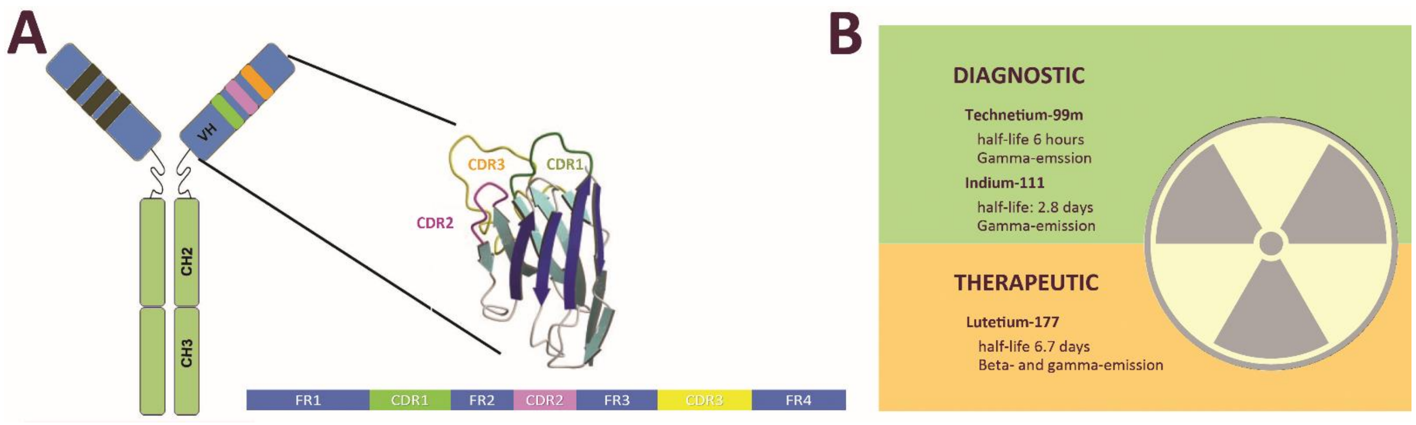

Supplementary Figure 1: (A) The antibody repertoire of camelids contains heavy-chain antibodies consisting of two heavy chains, lacking the additional light chains of conventional antibodies. The derived antigen-binding domain is a natural single-domain antibody with unique qualities. Its small size of around 15 kDa, its convex shape and the presence of a third complementarity determining region (CDR3) of unusual length allows a sdAb to bind structured, often cryptic epitopes that are frequently inaccessible to conventional antibodies. This variable part can be cloned and recombinantly produced. They consist of 3 CDRs and 4 framework regions (FR1–4). Its schematic representation (under) and conformation (upper part) of the sdAb are illustrated. (B) Illustration of the isotopes used in our experiments: radionuclides can have diagnostic or therapeutic capacities based on the emission of alpha- or beta-particles (resulting in ionizing radiation with therapeutic capacities) or the emission of gamma-rays or positrons (interesting for diagnosis by using SPECT or PET scintigraphy). Both^{99m}Tc and ^{111}In are gamma-emittors and can only used for the diagnosis. 177Lu emits beta-particles and gamma-rays: it is generally used as therapeutic agent, but its gamma-ray emission allows also tracing.

Supplementary Figure 2

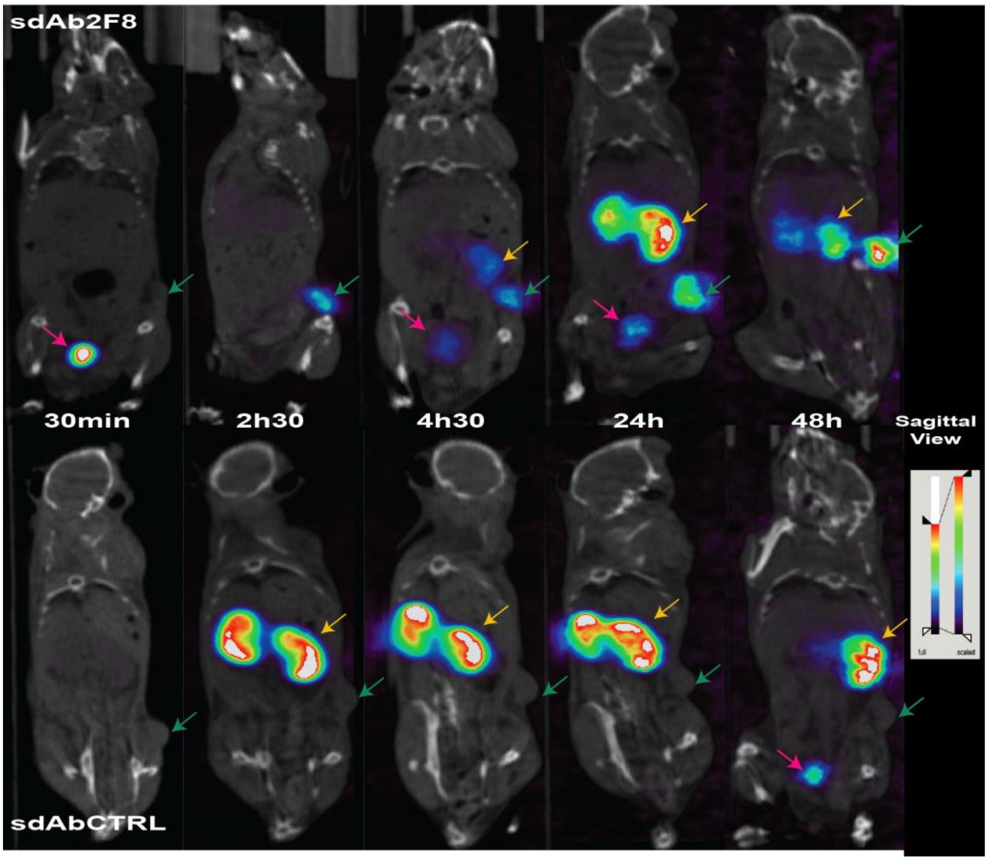

Supplementary Figure 2. Sagittal view on fused whole-body and tumour-focused μSPECT/CT acquisitions from 30min up to 48h p.i. of intravenously injected ^{111}In-DTPA-2F8 and non-targeting ^{111}In-DTPA-CTRL in cancer mice. One representative of each group at different time point are shown. Green arrows indicate tumour uptake, yellow ones indicate renal uptake while the pink arrows show the urinary bladder. NIH + white colour scale is used and images are normalised to the activity at the time of acquisition and equally scaled down to 70% relative to maximum activity in image.

Supplementary Figure 3

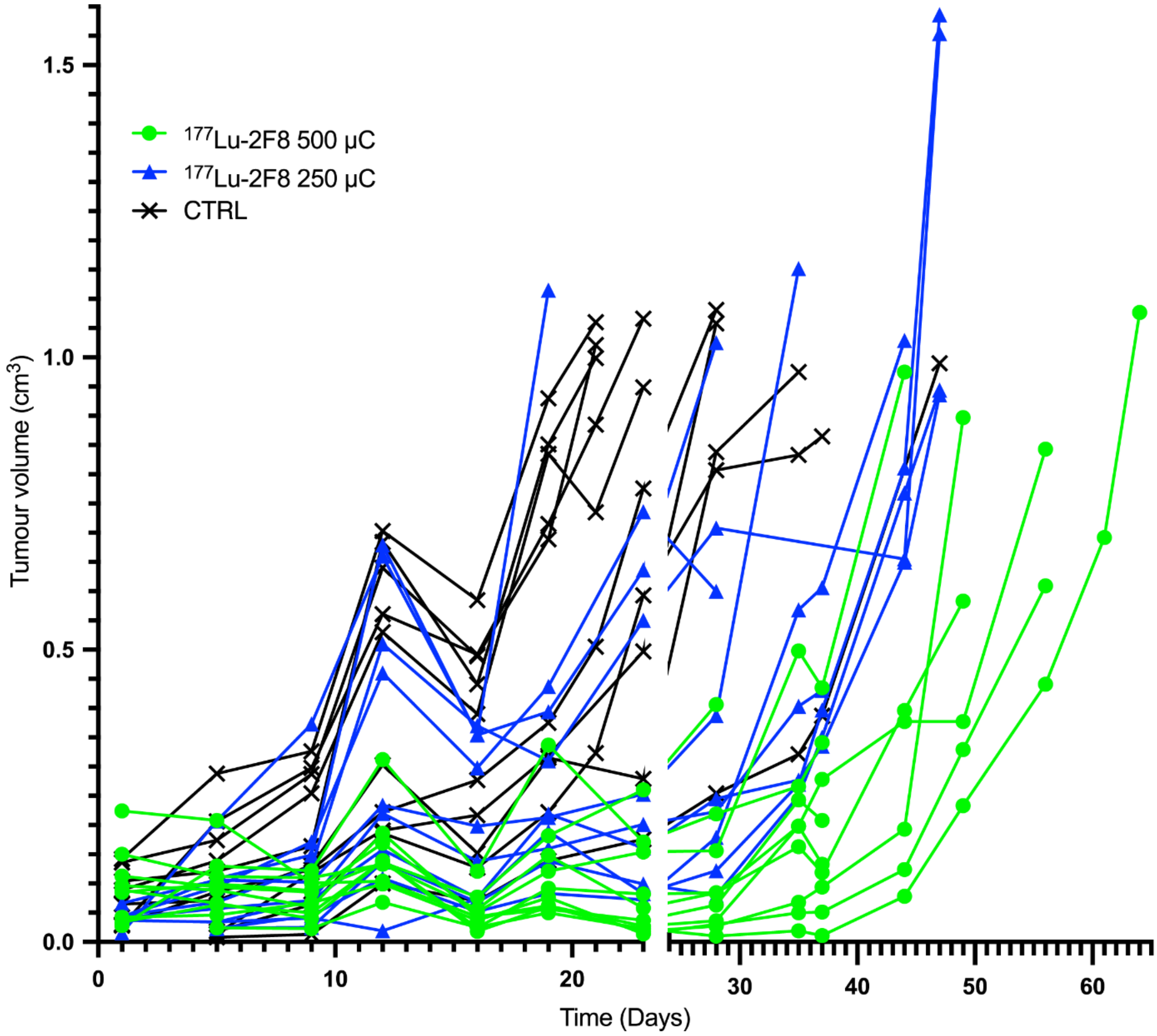

Supplementary Figure 3. Tumour volumes (cm^3) for individual mice (10 per group) in function of time during therapy. Mice were euthanised when tumour size exceeded $1cm^3$ or when > 15% weight loss was measured.

Supplementary table 1. Values of the *ex vivo* biodistribution of ^{99m}Tc-labelled sdAbs in RPMI 8226 CD38^{+} tumour bearing mice based on dissection data at 1h p.i. and expressed as % IA/g mean ± SD (n=3).

Tissue	sdAb 2F8	sdAb 551	sdAb 1053	sdAb CTRL
**Blood**	0.39 ± 0.09	0.61 ± 0.08	0.61 ± 0.26	1.02 ± 0.25
**Heart**	0.21 ± 0.03	0.33 ± 0.04	0.35 ± 0.16	0.61 ± 0.11
**Lung**	0.42 ± 0.25	0.57 ± 0.15	0.65 ± 0.26	1.13 ± 0.51
**Liver**	0.53 ± 0.12	1.33 ± 0.08	1.28 ± 0.28	1.16 ± 0.08
**Pancreas**	0.11 ± 0.02	0.18 ± 0.03	0.16 ± 0.06	0.28 ± 0.06
**Spleen**	0.18 ± 0.07	0.39 ± 0.08	0.25 ± 0.05	0.50 ± 0.06
**Right Kidney**	169.98 ± 5.37	403.87 ± 13.31	258.81 ± 19.51	271.76 ± 13.17
**Left Kidney**	168.65 ± 3.74	400.53 ± 31.61	257.19 ± 18.65	275.44 ± 23.59
**Stomach**	0.29 ± 0.04	0.30 ± 0.04	0.46 ± 0.09	0.62 ± 0.01
**Small Intestine**	0.23 ± 0.08	0.25 ± 0.04	0.34 ± 0.05	0.38 ± 0.16
**Large Intestine**	0.32 ± 0.11	0.22 ± 0.09	0.31 ± 0.08	0.48 ± 0.08
**Muscle**	0.26 ± 0.16	0.19 ± 0.04	0.18 ± 0.07	0.31 ± 0.07
**Bone**	0.22 ± 0.03	0.16 ± 0.06	0.23 ± 0.19	0.23 ± 0.08
**Tumour**	2.22 ± 0.47	3.37 ± 0.38	1.78 ± 0.37	0.79 ± 0.11
**Brain**	0.02 ± 0.01	0.03 ± 0.01	0.03 ± 0.01	0.05 ± 0.02

Supplementary table 2. Biodistribution analyses, 1h post-injection mice (^{99m}Tc-sdAbs) were sacrificed and tumours and organs were collected. Tumour-to-organ ratios are presented as mean ± SD (n=3).

Tissue	sdAb 2F8	sdAb 551	sdAb 1053	sdAb CTRL
**Blood**	5.66 ± 1.80	5.53 ± 0.95	2.94 ± 1.42	0.78 ± 0.22
**Heart**	10.44 ± 2.80	10.22 ± 2.31	5.00 ± 2.45	1.29 ± 0.30
**Lungs**	5.30 ± 3.36	5.92 ± 1.71	2.74 ± 1.25	0.70 ± 0.33
**Liver**	4.23 ± 1.36	2.54 ± 0.34	1.39 ± 0.42	0.68 ± 0.11
**Pancreas**	19.65 ± 4.93	18.74 ± 4.20	10.70 ± 4.51	2.83 ± 0.69
**Spleen**	12.15 ± 4.93	8.80 ± 2.18	7.04 ± 1.97	1.58 ± 0.29
**Kidneys**	0.01 ± 0.00	0.01 ± 0.00	0.01 ± 00	0.00 ± 0.00
**Stomach**	7.68 ± 2.03	11.12 ± 2.16	3.88 ± 1.09	1.27 ± 0.18
**Small Intestine**	9.41 ± 3.78	13.32 ± 2.51	5.35 ± 1.33	2.07 ± 0.90
**Large Intestine**	7.03 ± 2.96	15.10 ± 6.31	5.75 ± 1.88	1.65 ± 0.36
**Muscle**	8.68 ± 5.92	17.45 ± 4.54	9.73 ± 4.14	2.59 ± 0.73
**Bone**	9.82 ± 2.50	21.53 ± 8.43	7.87 ± 6.93	3.40 ± 1.26

Supplementary table 3. Comparative biodistribution of all four ^{99m}Tc-sdAbs injected in RPMI 8226 CD38^{+} NSG mice. Data, obtained from the regions of interest (ROI) drawn according to the organs on the SPECT/CT scans, are expressed as mean % injected activity/cc ± SD (n=3).

Tissue	sdAb 2F8	sdAb 1053	sdAb 551	sdAb CTRL
**Tumour**	1.74 ± 0.46	1.12 ± 0.26	1.29 ± 0.38	0.45 ± 0.05
**Liver**	0.35 ± 0.07	1.06 ± 0.24	0.80 ± 0.20	0.85 ± 0.08
**Heart**	0.34 ± 0.07	0.40 ± 0.11	0.49 ± 0.13	0.63 ± 0.11
**Left Kidney**	69.06 ± 2.26	91.18 ± 6.28	102.16 ± 7.82	94.88 ± 4.46
**Right Kidney**	58.46 ± 3.49	89.02 ± 11.62	77.83 ± 16.89	83.58 ± 9.44
**Lung**	0.24 ± 0.06	0.31 ± 0.09	0.33 ± 0.04	0.46 ± 0.06
**Muscle**	0.08 ± 0.01	0.11 ± 0.03	0.10 ± 0.04	0.13 ± 0.02

Supplementary table 4. Values of the *ex vivo* biodistribution of ^{111}In-labelled sdAbs co-injected or not with 150mg/kg of gelofusin in RPMI 8226 CD38^{+} tumour bearing mice based on dissection data at 1h p.i. and expressed as % IA/g mean ± SD (n=3).

Tissue	sdAb 2F8 + Gelo	sdAb 2F8	sdAb CTRL
**Blood**	0.52 ± 0.07	0.48 ± 0.22	0.47 ± 0.15
**Heart**	0.16 ± 0.02	0.27 ± 0.11	0.24 ± 0.04
**Lung**	0.40 ± 0.10	0.54 ± 0.33	0.98 ± 0.94
**Liver**	0.66 ± 0.14	0.41 ± 0.03	0.38 ± 0.10
**Pancreas**	0.20 ± 0.05	0.22 ± 0.11	0.24 ± 0.02
**Spleen**	0.14 ± 0.02	0.23 ± 0.14	0.17 ± 0.01
**Right Kidney**	12.92 ± 0.02	22.99 ± 2.62	17.18 ± 3.33
**Left Kidney**	12.30 ± 0.05	24.77 ± 11.12	16.93 ± 1.90
**Stomach**	0.41 ± 0.07	0.28 ± 0.02	0.29 ± 0.13
**Small Intestine**	0.27 ± 0.01	0.28 ± 0.03	0.26 ± 0.07
**Large Intestine**	0.38 ± 0.12	0.34 ± 0.15	0.36 ± 0.03
**Muscle**	0.16 ± 0.04	0.16 ± 0.08	0.17 ± 0.07
**Bone**	0.20 ± 0.02	0.23 ± 0.07	0.27 ± 0.07
**Tumour**	3.08 ± 1.47	3.38 ± 0.42	0.54 ± 0.14

Supplementary table 5. Biodistribution (from 30min up to 48h) of ^{111}In-sdAb 2F8 injected in combination with gelofusin (150m/kg) in mice bearing RPMI CD38^{+} tumour killed 48h p.i. Data, obtained from the regions of interest (ROI) drawn according to the organs on the SPECT/CT scans, are expressed as mean % injected activity/cc ± SD (n=3).

Tissue	30min	2h30	4h30	24h	48h
Tumour	1.06 ± 0.06	1.2 ± 0.15	1.04 ± 0.33	0.78 ± 0.32	0.76 ± 0.16
Left Kidney	3.63 ± 1.06	5.12 ± 0.73	4.72 ± 0.52	2.02 ± 0.15	1.14 ± 0.13
Right Kidney	3.93 ± 1.26	5.68 ± 0.83	5.44 ± 0.87	2.43 ± 0.18	1.22 ± 0.06
Liver	1.17 ± 1.09	0.76 ± 0.88	0.66 ± 0.74	0.24 ± 0.22	0.13 ± 0.03
Lungs	0.47 ± 0.16	0.12 ± 0.06	0.14 ± 0.13	0.03 ± 0.00	0.05 ± 0.02
Muscle	0.26 ± 0.06	0.05 ± 0.01	0.04 ± 0.01	0.03 ± 0.01	0.02 ± 0.00
Heart	0.62 ± 0.16	0.08 ± 0.03	0.08 ± 0.02	0.03 ± 0.01	0.03 ± 0.01

Supplementary table 6. Biodistribution (from 30min up to 48h) of non-specific ^{111}In-sdAb CTRL and injected in combination with gelofusin (150m/kg) in mice bearing RPMI CD38^{+} tumour killed 48h p.i. Data, obtained from the regions of interest (ROI) drawn according to the organs on the SPECT/CT scans, are expressed as mean % injected activity/cc ± SD (n=3).

Tissue	30min	2h30	4h30	24h	48h
Tumour	0.52 ± 0.14	0.16 ± 0.02	0.13 ± 0.02	0.05 ± 0.01	0.06 ± 0.02
Left Kidney	7.17 ± 0.66	9.42 ± 0.50	7.63 ± 0.49	2.55 ± 0.18	1.32 ± 0.10
Right Kidney	7.77 ± 0.09	8.42 ± 0.53	6.69 ± 0.35	2.45 ± 0.17	1.27 ± 0.02
Liver	0.43 ± 0.05	0.21 ± 0.11	0.17 ± 0.11	0.12 ± 0.05	0.10 ± 0.03
Lungs	0.44 ± 0.08	0.07 ± 0.01	0.05 ± 0.01	0.04 ± 0.01	0.03 ± 0.01
Muscle	0.39 ± 0.11	0.08 ± 0.02	0.06 ± 0.01	0.04 ± 0.01	0.04 ± 0.01
Heart	0.48 ± 0.08	0.05 ± 0.01	0.06 ± 0.01	0.03 ± 0.02	0.02 ± 0.00

Supplementary table 7. Values of the *ex vivo* biodistribution of ^{177}Lu-labelled sdAbs co-injected with 150mg/kg of gelofusin in RPMI 8226 CD38^{+} tumour bearing mice based on dissection data at different time point up to 48h p.i. and expressed as % IA/g mean ± SD (n=3).

Tissue	sdAb 2F8 1H p.i.	sdAb 2F8 3H p.i.	sdAb 2F8 6H p.i.	sdAb 2F8 24H p.i.	sdAb 2F8 48H p.i.	Dose (cGy/MBq)
**Blood**	0.33 ± 0.06	0.02 ± 0.001	0.01 ± 0.001	0.01 ± 0.001	0.002 ± 0.001	0.06
**Heart**	0.23 ± 0.03	0.07 ± 0.01	0.07 ± 0.001	0.04 ± 0.000	0.04 ± 0.009	0.17
**Lung**	0.41 ± 0.05	0.14 ± 0.02	0.14 ± 0.05	0.05 ± 0.01	0.03 ± 0.01	0.31
**Liver**	0.35 ± 0.05	0.19 ± 0.02	0.19 ± 0.03	0.09 ± 0.01	0.08 ± 0.01	0.41
**Pancreas**	0.19 ± 0.02	0.08 ± 0.004	0.08 ± 0.01	0.05 ± 0.01	0.05 ± 0.01	0.19
**Spleen**	0.26 ± 0.03	0.13 ± 0.003	0.12 ± 0.02	0.10 ± 0.03	0.09 ± 0.03	0.37
**Right Kidney**	18.36 ± 0.72	14.98 ± 1.21	13.05 ± 1.60	2.00 ± 0.27	1.22 ± 0.46	22.18
**Left Kidney**	18.41 ± 0.34	13.60 ± 0.43	12.48 ± 1.50	2.08 ± 0.26	1.21 ± 0.47	
**Stomach**	0.52 ± 0.05	0.50 ± 0.32	0.21 ± 0.17	0.11 ± 0.04	0.05 ± 0.002	0.59
**Small Intestine**	0.38 ± 0.12	0.13 ± 0.04	0.08 ± 0.01	0.06 ± 0.01	0.03 ± 0.01	0.25
**Large Intestine**	0.40 ± 0.11	0.28 ± 0.15	0.38 ± 0.30	0.28 ± 0.13	0.25 ± 0.06	1.10
**Muscle**	0.33 ± 0.18	0.03 ± 0.004	0.03 ± 0.01	0.02 ± 0.004	0.02 ± 0.01	0.10
**Bone**	0.41 ± 0.30	0.07 ± 0.01	0.05 ± 0.01	0.07 ± 0.01	0.07 ± 0.02	0.22
**Tumour**	4.51 ± 0.29	3.32 ± 0.25	3.69 ± 0.16	2.36 ± 0.49	3.46 ± 1.33	9.91
